# Increase of leishmanicidal and tubercular activities using steroids linked to aminoquinoline

**DOI:** 10.1186/2191-2858-2-16

**Published:** 2012-05-02

**Authors:** Luciana MR Antinarelli, Arturene ML Carmo, Fernando R Pavan, Clarice Queico F Leite, Adilson D Da Silva, Elaine S Coimbra, Deepak B Salunke

**Affiliations:** 1 Departamento de Parasitologia, Microbiologia e Imunologia, I.C.B, Universidade Federal de Juiz de Fora, Campus Universitário, Juiz de Fora, MG 36036-900, Brazil; 2 Departamento de Química, I.C.E., Universidade Federal de Juiz de Fora, Campus Universitário, Juiz de Fora, MG 36036-900, Brazil; 3 Departamento de Ciências Biológicas, F.C. F., Universidade Estadual Paulista “Júlio de Mesquita Filho”, Rod. Araraquara-Jaú Km.01 s/n, Campus, 14801-902, Araraquara, SP, Brazil; 4 Centre National de la Recherche Scientifique, Institut de Chimie des Substances Naturelles, UPR 2301 CNRS, Avenue de la Terrasse, 91198, Gif-sur-Yvette, France

**Keywords:** Antileishmanial drugs, Antituberculosis drugs, Click chemistry, Quinoline, Steroid

## Abstract

**Background:**

Aminoquinoline/steroid conjugates were synthesized based on the fact that steroid transporters have been shown to accept and carry a variety of drugs. So, in continuing our research of antileishmanial and antitubercular drugs, aminoquinoline/steroid conjugates (**12**, **13,** and **14)** were regioselectively synthesized via 1, 3-dipolar cycloaddition of alkynes **3, 5**, and **7** with azide **12**. The aminoquinoline/steroids conjugates were evaluated *in vitro* against *Leishmania major* and *Mycobacterium tuberculosis*.

**Results:**

Regioselective synthesis of the novel aminoquinoline/steroid conjugates was achieved in very high yield. All aminoquinoline/steroid conjugates (**12**, **13,** and **14**) exhibited best results against *Leishmania* and *M. tuberculosis* than the respective alkyne intermediate structures (**3**, **5,** and **7**, respectively). Among them, the compound **12** exhibited the best activity for *M. tuberculosis* (MIC = 8.8 μM). This result is comparable to drugs commonly used in tuberculosis treatment. Also, for antileishmanial assay, the aminoquinoline/steroid conjugates demonstrated a significant activity against promastigote and amastigote forms of *L. major*.

**Conclusions:**

Addition of a steroid group to aminoquinoline molecules enhanced the leishmanicidal and antitubercular activities. These results highlight the importance of steroids as carrier.

## Background

Quinolines are among the most important antimalarial drugs ever used [1,2]. In addition, quinoline derivatives have also demonstrated a variety of biological properties that includes antiviral, anti-inflammatory, antitubercular, and antileishmanial activities [2-5]. Leishmaniasis is a disease caused by parasitic protozoans of the genus *Leishmania*. Over 20 different *Leishmania* species can infect humans and cause a wide spectrum of symptoms. It has an estimated prevalence of 12 million cases worldwide, which is continuing to increase, with 1.5–2 million new cases each year [6]. With no available vaccine, the chemotherapy is a major control for the disease. However, the treatment options are severely limited and first line treatment is based on pentavalent antimonials that have been used in therapeutics for more than half a century [7]. Tuberculosis (TB) is another important neglected disease. TB is more prevalent in the world today than at any other time in human history. *Mycobacterium tuberculosis* (MTB), the pathogen responsible for TB, uses diverse strategies to survive in a variety of host lesions and to evade immune surveillance [7,8]. The last 20 years have seen the worldwide appearance of multidrug-resistant TB, followed by extensively drug-resistant TB, and most recently, strains that are resistant to all antituberculosis drugs [9]. Since the discovery of rifampicin (1960), no new drugs have been developed specifically against mycobacteria [10]. Also, only within the last few years some promising drug candidates have emerged [11]. Considering the inefficacy and the high toxicity of the currently used drugs for the treatment of these infectious diseases, as well as the emergence of drug-resistant strains of the causative organisms, the development of new leishmanicidal and antitubercular agents is extremely important.

Bioconjugation has emerged as a fast growing technology and aims at the ligation of two or more molecules to form new complexes with the combined properties of their individual components [12]. To make this linkage, the 1,2,3-triazole moieties are attractive as connecting units, since they are stable to metabolic degradation and capable of hydrogen bonding, which can be favorable in binding to biomolecular targets and also improves solubility [13]. Although the 1,2,3-triazole structural moiety does not occur in nature, the synthetic molecules containing the 1,2,3-triazole unit show diverse biological activities including antibacterial, herbicidal, fungicidal, anti-allergic, and anti-HIV [14]. Aminoquinoline/cholic acid conjugates were synthesized based on the fact that steroid transporters have been shown to accept and carry a variety of drugs [15]. Cholic acid is the most common form of the steroid and its derivatives are known to exhibit antimicrobial activities [16]. Bile acids are amphiphilic molecules which may represent alternatives for chemotherapeutic agents by acting synergistically with antibiotics as membrane permeabilizers [17-21]. Moreover, several bile acid/drug conjugates are shown to possess better activity than the precursor [22,23].

In a previous study, we demonstrate that 4-amino-7-chloroquinoline derivatives showed an interesting antileishmanial and anti-MTB activities [24]. In continuation of this study were synthesized aminoquinoline conjugate with steroids in the expectation of improving its biological activity.

## Methods

### General experimental techniques and apparatus

TLC was performed on precoated silica gel F254 plates (0.25 mm; E. Merck). Infrared spectra were recorded on Schimadzu 8400 series FTIR instrument. ^1^ H NMR spectra were recorded on a Bruker AC-300 and 500 spectrometers at 300.13 and 500.13 MHz and ^13^C NMR spectra were recorded on a Bruker AC-300 at 75 MHz. The chemical shifts are given in parts per million relative to tetramethylsilane. Mass spectra were recorded on LC–MS/MS-TOF API QSTAR PULSAR spectrometer, and samples were introduced by infusion method using Electro spray Ionization Technique. Standard work up: after extraction of all the reactions, the organic extracts were washed with water and brine and dried over anhydrous Na_2_SO_4_ and concentrated in vacuum.

### Synthesis of terminal acetylenes

#### General synthetic procedure for N-(7-chloroquinolin-4-yl)alkyl-diamine) (2, 4, and 6)

A mixture of 4,7-dichloroquinoline (2.0 g, 10.1 mmol) and the corresponding diamines (50.0 mmol), ethane-1,2-diamine, propane-1,3-diamine, or buthane-1,4-diamine, was heated at 80°C for 1 h without stirring for 1 h and then at 110°C for 4–6 h with continued stirring to drive the reaction to completion. The reaction mixture was cooled to room temperature and diluted with dichloromethane. The organic layer was successively washed with 5% NaOH (30 mL) followed by water wash and then finally with brine. The organic layer was dried over anhydrous Na_2_SO_4_ and solvent was removed under reduced pressure to afford the compounds **2**, **4,** and **6**, at 80–90% yield.

*N-(7-chloroquinolin-4-yl)ethane-1,2-diamine (****2****)*[25]: Yellow solid, yield: 90%; mp = 141°C (145-147°C).

*N-(7-chloroquinolin-4-yl)propane-1,3-diamine (****4****)*[25]: Yellow solid, yield: 90%; mp = 97°C (96–98°C) [25].

*N-(7-chloroquinolin-4-yl)buthane-1,4-diamine (****6****)*[25]: Yellow solid, yield: 80%; mp = 123°C (122–124°C).

#### General synthetic procedure for 7-chloro-N-(3-(prop-2-ynylamino)alquil)quinolin-4-amine (3, 5, and 7)

The compounds **2**, **4,** and **6** (6.8 mmol) and propargyl bromide (13.6 mmol), in presence of K_2_CO_3_ (13.6 mmol), were dissolved in EtOH (5.0 mL). The reaction mixture was stirred at 0°C for 2 h and then at 25°C for 48 h. Solvent was removed in vacuum until dry. The crude reaction product was purified by flash chromatography (eluent: MeOH/CH_2_Cl_2_ 5:95) producing the compounds **3, 5,** and **7,** respectively (2.5 mmol) in 60% yield as yellow solid.

*7-chloro-N-(2-(prop-2-ynylamino)ethyl)quinolin-4-amine (****3****)*[24]: Yield: 60%, mp = 99°C.

*7-chloro-N-(3-(prop-2-ynylamino)propyl)quinolin-4-amine (****5****)*[24]: Yield: 60%, mp = 75°C.

*7-chloro-N-(4-(prop-2-ynylamino)butyl)quinolin-4-amine (****7****)*[24]: Yield: 62%, mp = 72°C.

### Synthesis of terminal azide

#### *Synthesis of methyl 3α,7α,12α-trihydroxy-5β-cholane-24-oate (9)*

Compound **9** was synthesized in overall good yield starting from bile acid **8** using the literature procedure [24]. White solid, m.p. 158°C.

*Methyl-3α-mesyloxy-7α-12α-dihydroxy-5β-cholane-24-oate (****10****)*[23]: To a solution of **9** (2.0 g, 4.92 mmol) in CH_2_Cl_2_ (20 mL) was added triethylamine (6.4 mL, 49.2 mmol) at 0°C. Methane sulfonyl chloride (0.5 mL, 4.92 mmol) was added dropwise for 10 min at 0°C. The reaction mixture was extracted with CH_2_Cl_2_/H_2_O. Organic layer was washed with NaHCO_3_, water, and brine. The solvent was evaporated under reduced pressure. The crude product was purified by column chromatography (AcOEt/Hex 2:8) to obtain pure product **7** (1.9 g).

#### Synthesis of methyl-3β-azido-7α,12α-dihydroxy-5β-cholane-24-oate (11)

The compound **10** was reacted with NaN_3_ (5 equiv) in DMF for 24 h at 120°C to give product **11**[23]. White solid, m.p. 175°C.

#### General procedure for cycloaddition (12–14)

The alkyne **3**, **5,** or **7** (1 equiv) and the azide **11** (1.3 equiv) were dissolved in DMSO/H_2_O 4:1 (5 mL). To this solution, CuSO_4_.5H_2_O (0.05 equiv) and sodium ascorbate (0.40 equiv) were added. The reaction mixture was stirred for 48 h at room temperature and it was then extracted with CH_2_Cl_2_/H_2_O. Organic layer was washed with NaHCO_3_, water, and brine. The solvent was evaporated under reduced pressure and crude product was purified by column chromatography on silica gel using 30% MeOH/CH_2_Cl_2_ system to obtain aminoquinoline/bile acid conjugates **12, 13,** or **14,** respectively, linked with 1,4-disubstituted 1,2,3-triazole in 60% yield.

*Methyl 3β-(N-[(7-chloroquinolin-4-yl)amino]ethylaminomethyl)-1 H-1,2,3-triazol-1-yl)]7α-12α-dihydroxy-5β-cholane-24-oate (****12****)*: Yellow crystalline solid; m.p. 128°C, υ_max_ (KBr): 3340 (NH), 2930 (CH); ^1^ H NMR (300 MHz, CD_3_OD): 8.31 (d, 1 H, *J*_2,3_ = 4 Hz, H-2’); 8.08 (d, 1 H, *J*_5,6_ = 6 Hz, H-5’); 7.88 (s, 1 H, H-4” triazole); 7.73 (s, 1 H, H-8’); 7.37 (dd, 1 H, *J*_6,5_ = 6 Hz, *J* = 2 Hz, H-6’); 6.51 (d, 1 H, *J*_3,2_ = 4 Hz, H-3’); 4.53 (s, 1 H, H-12); 3.60 (s, 1 H, H-7); 3.89 (s, 2 H, H-3”); 3.49 (s, 3 H, H-25); 3.45 (m, 2 H, H-1”); 2.93 (m, 2 H, H-2”); 0.97 (d, 3 H, *J* = 6 Hz, H-21); 0.76 (s, 3 H, H-18); 0.65 (s, 3 H, H-19); ^13^C NMR (75 MHz, CD_3_OD): 176.4 (C-24); 152,6 (C-4’); 152.0 (C-2’); 149.3 (C-9’); 146.3 (C-3” triazole); 136.3 (C-7’); 127.3 (C-8’); 125.9 (C-6’); 124.3 (C-4” triazole); 123.3 (C-5’); 118.6 (C-10’); 99.5 (C-3’); 73.7 (C-12); 68.7 (C-7); 58.2 (C-3); 51.8 (C-13); 48.8 (C-25); 47.1 (C-2); 23.3 (C-21); 17.4 (C-19); 12.8 (C-18); HRMS ESI [M + H]^+^: m/z: Calc for C_39_H_56_N_6_O_4_Cl 707.4052 [M + H]^+^, found 707.4059 [M + H]^+^.

*Methyl 3β-(N-[(7-chloroquinolin-4-yl)amino]propylaminomethyl)-1 H-1,2,3-triazol-1-yl)]7α-12α-dihydroxy-5β-cholane-24-oate (****13****)*: Yellow oil; υ_max_ (KBr): 3345 (NH), 2928 (CH); ^1^ H NMR (300 MHz, CDCl_3_): 8.38 (d, 1 H, *J*_2,3_ = 4 Hz, H-2’); 7.87 (s, 1 H, H-8’); 7.74 (d, 1 H, *J*_5,6_ = 6 Hz, H-5’); 7.52 (s, 1 H, H-5” triazole); 7.20 (dd, 1 H, *J*_6,5_ = 6 Hz, *J* = 2 Hz, H-6’); 6.27 (d, 1 H, *J*_3,2_ = 4 Hz, H-3’); 4.53 (s, 2 H, H-4”); 3.87 (s, 1 H, H-7); 3.65 (s, 3 H, H-25); 3.45 (m, 2 H, H-1”); 0.97 (d, 3 H, *J* = 6 Hz, H-21); 0.81 (s, 3 H, H-18); 0.68 (s, 3 H, H-19); ^13^C NMR (75 MHz, CDCl_3_): 174.9 (C-24); 151.4 (C-4’); 150.2 (C-2’); 144.6 (C-9’); 144.6 (C-4” triazole); 135.5 (C-7’); 126.8 (C-8’); 125.4 (C-6’); 123.0 (C-5” triazole); 122.9 (C-5’); 121.4 (C-10’), 114.0 (C-3’), 73.0 (C-12), 68.2 (C-7), 57.0 (C-3), 51.7 (C-13), 48.4 (C-25), 47.4 (C-2), 38.3 (C-14); 22.9 (C-21); 17.5 (C-19); 12.7 (C-18); HRMS ESI [M + H]^+^: m/z: Calc for C_40_H_58_N_6_O_4_Cl 721.4108 [M + H]^+^, found 721.4210 [M + H]^+^.

*Methyl 3β-(N-[(7-chloroquinolin-4-yl)amino]buthylaminomethyl)-1 H-1,2,3-triazol-1-yl)]7α-12α-dihydroxy-5β-cholane-24-oate (****14****)*.

Yellow oil; υ_max_ (KBr): 3347 (NH), 2931 (CH); ^1^ H NMR (300 MHz, CDCl_3_): 8.44 (d, 1 H, *J*_2,3_ = 2 Hz, H-2’); 7.88 (s, 1 H, H-8’); 7.77 (d, 1 H, *J*_5,6_ = 6 Hz, H-5’); 7.51 (s, 1 H, H-7” triazol); 7.23 (dd, 1 H, *J*_6,5_ = 6 Hz, *J* = 2 Hz, H-6’); 6.32 (d, 1 H, *J*_3,2_ = 2 Hz, H-3’); 3.92 (s, 2 H, H-4”); 3.88 (s, 1 H, H-7); 3.66 (s, 3 H, H-25); 3.28 (m, 2 H, H-1”); 0.99 (d, 3 H, *J* = 6 Hz, H-21); 0.82 (s, 3 H, H-18); 0.68 (s, 3 H, H-19); ^13^C NMR (75 MHz, CDCl_3_): 174.9 (C-24); 151.6 (C-4’); 150.5 (C-2’); 148.7 (C-9’); 145.3 (C-6” triazole); 134.8 (C-7’); 127.8 (C-8’); 124.9 (C-6’); 122.3 (C-7” triazole); 121.2 (C-5’); 117.4 (C-10’); 98.8 (C-3’); 72.9.0 (C-12); 68.0 (C-7); 56.9 (C-3); 51.6 (C-13); 48.7 (C-25); 47.3 (C-2); 22.9 (C-21); 17.5 (C-19); 12.7 (C-18); HRMS ESI [M + H]^+^: m/z: Calc for C_41_H_60_N_6_O_4_Cl 735.4365 [M + H]^+^, found 735.4362 [M + H]^+^.

### Biological evaluation

#### Anti-MTB activity

The anti-MTB activity of the compounds was determined by the *Resazurin Microtiter Assay* (REMA) [26]. Stock solutions of the test compounds were prepared in dimethyl sulfoxide (DMSO) and diluted in Middlebrook 7 H9 broth (Difco), supplemented with oleic acid, albumin, dextrose and catalase (OADC enrichment—BBL/Becton Dickinson, Sparks, MD, USA), to obtain final drug concentration ranges from 0.15 to 250 μM. The serial dilutions were realized in a Precision XS Microplate Sample Processor (Biotek™). The isoniazid was dissolved in distilled water, as recommended by the manufacturer (Difco laboratories, Detroit, MI, USA), and used as a standard drug. MTB H_37_Rv ATCC 27294 was grown for 7 to 10 days in Middlebrook 7 H9 broth supplemented with OADC, plus 0.05% Tween 80 to avoid clumps. Cultures were centrifuged for 15 min at 3,150 *g*, washed twice, and resuspended in phosphate-buffered saline and aliquots were frozen at −80°C. After 2 days, an aliquot was thawed to determine the viability and the CFU after freezing. MTB H_37_Rv (ATCC 27294) was thawed and added to the test compounds, yielding a final testing volume of 200 μL with 2 × 10^4^ CFU/mL. Microplates with serial dilutions of each compound were incubated for 7 days at 37°C, after resazurin was added to test viability. Wells that turned from blue to pink, with the development of fluorescence, indicated growth of bacterial cells, while maintenance of the blue color indicated bacterial inhibition [26]. The fluorescence was read (530 nm excitation filter and 590 nm emission filter) in a SPECTRAfluor Plus (Tecan^®^) microfluorimeter. The MIC was defined as the lowest concentration resulting in 90% inhibition of growth of MTB. As a standard test, the MIC of isoniazid was determined on each microplate. The acceptable range of isoniazid MIC is from 0.11 to 0.44 μM [10,33]. Each test was set up in triplicate.

### *In vitro* antileishmanial activity

#### Parasites and cell culture

Promastigote forms of *L. major* (MRHO/SU/59/P) were maintained in Medium BHI supplemented with 10% fetal bovine serum (FBS) at 24°C. FBS was purchased from Cultilab (Campinas, São Paulo, Brazil) and brain heart infusion (BHI) from Himédia (Mumbai, India).

#### Promastigote forms

The viability of parasites was determined by the colorimetric 3-(4,5-dimethylthiazol-2-yl)-2,5-diphenyl-tetrazolium bromide (purchased by Sigma Chemical Co., St. Louis, MO, USA) or MTT method, based on tetrazolium salt reduction by mitochondrial dehydrogenases [27]. Briefly, promastigotes of *L. major* from a logarithmic phase culture were suspended to yield 2 million cells/mL after Neubauer chamber counting. The screening was performed in 96-well microtiter plates maintained at 24°C. Controls with DMSO and without drugs were performed. Absorbance was measured at 570 nm (Multiskan MS microplate reader, LabSystems Oy, Helsink, Finland). The results are expressed as the concentrations inhibiting parasite growth by 50% (IC_50_) after a 3-day incubation period. Amphotericin B (supplied by Cristália, São Paulo, Brazil) was used as the reference standard. For data analysis: IC_50_ values were carried out at 5% significance level (*p* < 0.05, CI 95%), calculated using a nonlinear regression curve, by using *GraFit* Version 5 software (Erithacus Software Ltd., Horley, UK).

#### Amastigote forms

Concerning the amastigotes *in vitro* model, inflammatory macrophages were obtained from BALB/c mice previously inoculated with 3% thioglycollate medium (Sigma Chemical Co.). Briefly, peritoneal macrophages were plated at 2 × 10^6^ cells/mL on coverslips (13-mm diameter) previously arranged in a 24-well plate in RPMI 1640 medium supplemented with 10% inactivated FBS, and allowed to adhere for 24 h at 37°C in 5% CO_2_. Adherent macrophages were infected with *L. major* (MRHO/SU/59/P) promastigotes in the stationary growth phase using a ratio of 1:10 at 37°C for 3 h. Non-internalized promastigotes were eliminated and solutions of tested compounds were added and maintained at 37°C in 5% CO_2_ for 72 h. Slides were fixed and stained with Giemsa for parasite counting (optical microscopy, 1000× magnification). Amphotericin B was used as a standard drug and the reduction of the number of amastigotes was evaluated after only 24-h post-infection (0.1 μM = 35% and 1.0 μM = 48% of reduction of intracellular amastigotes). The data were analyzed using GraphPad Prism 5.0 (GraphPad Software, San Diego, CA, USA), which considered the mean of two assays performed in duplicate. One-way ANOVA was applied to compare all the groups. Differences were regarded as significant when *p* < 0.0001 (***) and *p* < 0.001 (**).

## Results and discussion

### Chemistry

**Figure 1:**
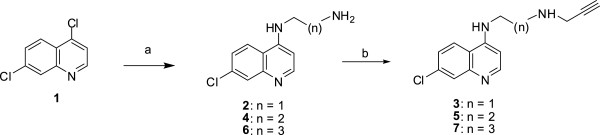
**Reagents and conditions: (a) ethane-1,2-diamine, propane-1,3-diamine, or butane-1,4-diamine, 80°C for 1 h, 110°C for 4 h, 90%; (b) propargyl bromide, K**_**2**_**CO**_**3**_**, EtOH, 25°C, 48 h, 60%.**

The aminoquinoline/steroids conjugates **12**, **13**, **and 14** were synthesized via 1,3-dipolar cycloaddition of alkyne **3**, **5,** or **7**, respectively, with an azide group of the bile acid **11**. 4,7-dichloroquinoline **1** on treatment with ethylenediamine, propanediamine, or butanediamine at 80–110°C for 4 h furnished the intermediates *N*-(2-aminoethyl)-7-chloroquinolin-4-amine (**2**), *N*-(3-aminopropyl)-7-chloroquinolin-4-amine (**4**), and *N*-(4-aminobutyl)-7-chloroquinolin-4-amine (**6**) in 90% yield [25]. These intermediates **2**, **4,** or **6** on further treatment with propargyl bromide and K_2_CO_3_ in EtOH at 25°C for 48 h yielded compounds **3**, **5,** and **7**, respectively, in 60% yield (see Figure 1) [28].

**Figure 2:**
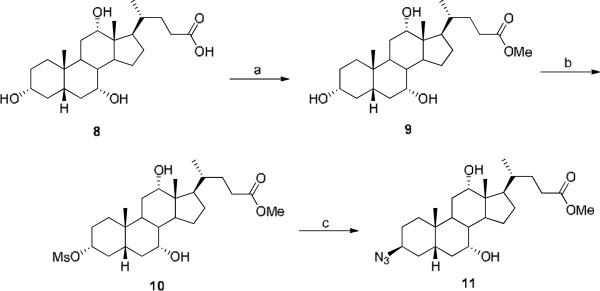
**Reagents and conditions: (a) HCl/MeOH, 25°C, 24 h, 98%; (b) MsCl, Et**_**3**_**N, CH**_**2**_**Cl**_**2**_**, 0°C, 2 h, 80%; (c) NaN**_**3**_**, DMF, 120°C, 24 h, 70%.**

**Figure 3:**
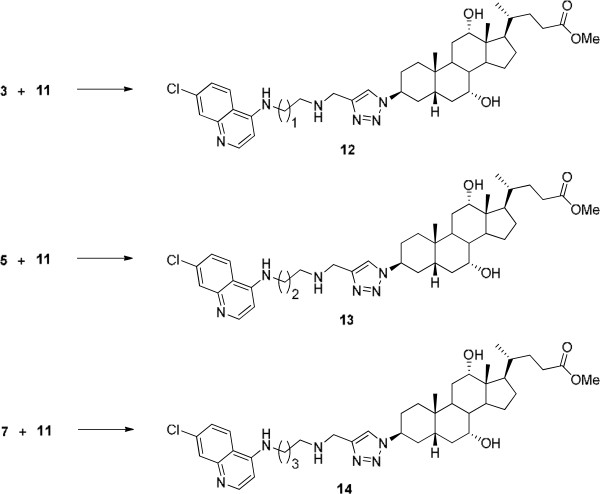
**Reagents and conditions: (a) CuSO**_**4**_**.5H**_**2**_**O, sodium ascorbate, DMSO/H**_**2**_**O (1:1), 25°C, 96 h, 60%.**

The C-3-azido steroid (bile acid) derivative **11** was synthesized according to the literature procedures [29,30] with small modifications (see Figure 2). Finally, the aminoquinoline/steroid (bile acid) conjugates **12**, **13**, and **14** were synthesized in very high yield via 1,3-dipolar cycloaddition of alkyne **3**, **5,** or **7** with an azide group of the bile acid **11**, respectively, using CuSO_4_·5H_2_O, sodium ascorbate, DMSO/H_2_O (1:1) at 25°C for 96 h, in 60% yield (see Figure 3). All the compounds were well characterized by ^1^ H NMR, ^13^C NMR, and HRMS.

### Biological evaluation

Previous study demonstrated that 4-amino-7-chloroquinoline derivatives (**2–7**) showed an interesting antileishmanial and anti-MTB activities [24]. In continuation of this study, novel steroid linked aminoquinolines were synthesized in an anticipation to improve its biological activity. Table 1 shows the biological results comparing the alkyne intermediate structures (**3**, **5,** and **7**) and their corresponding aminoquinoline/steroid conjugate products (**12**, **13,** and **14)**.

**Table 1:** **Effect of the compounds on promastigote forms of *L. major, *murine peritoneal macrophages and *M. tuberculosis***

**Compounds**	**Biological tests (μM)**	
	***M. tuberculosis *(MIC)**^**a**^	***L. major *(IC**_**50**_**)**^**b**^
**3**	60.1^c^	20.6^c^
**5**	60.1^c^	45.0^c^
**7**	54.2^c^	>87.0^c^
**12**	8.8	10.6
**13**	17.3	21.2
**14**	17.0	25.6
AmB*	–	0.3
Isoniazid*	0.11–0.44	–

Values represent the mean of triplicate samples.*AmB (amphotericin B) and isoniazid were used as reference drug for antileishmanial and anti-MTB assays, respectively. ^a^IC_50_ values (concentrations inhibiting parasite growth by 50%). ^b^MIC: lowest concentration resulting in 90% inhibition of growth of MTB. ^c^Data have been reported previously [24].

Anti-MTB activity of the compounds increased in the following order: alkyne intermediate structures (**3**, **5,** and **7**) < aminoquinoline/steroid conjugates (**12**–**14**). The aminoquinoline/steroid conjugates (**12**–**14**) showed excellent results with MICs ranging from 8.8 to 17.3 μM. Within these conjugates, the compound **12** was the most active against MTB bacilli (8.8 μM) and the presence of the shortest ethylenodiamine linker was enough to demonstrate the improved activity. The minimum inhibitory concentration (MIC) value found for the compound **12** is comparable or better than the MIC of some “second-line” drugs currently used in TB therapy such as cycloserine (122.4–489.7 μM), kanamycin (2.1–8.6 μM), tobramycin (8.6–17.1 μM), and clarithromycin (10.7–21.4 μM) [31].

For antileishmanial test, the assay was performed in both promastigote and amastigote forms of *Leishmania* since both stages of parasite are used for drug screening research [32-34]. Table 1 shows IC_50_ values of synthesized compounds on promastigote forms of *L. major*. Aminoquinoline/steroid conjugates (**12**–**14**) were more active than the respective alkyne intermediate structures (**3**, **5,** and **7**, respectively). Among them, the compound **12** was the most active in promastigotes of *L. major*, inhibiting two times more the viability of the parasite than the alkyne intermediate **3**.

**Figure 4:**
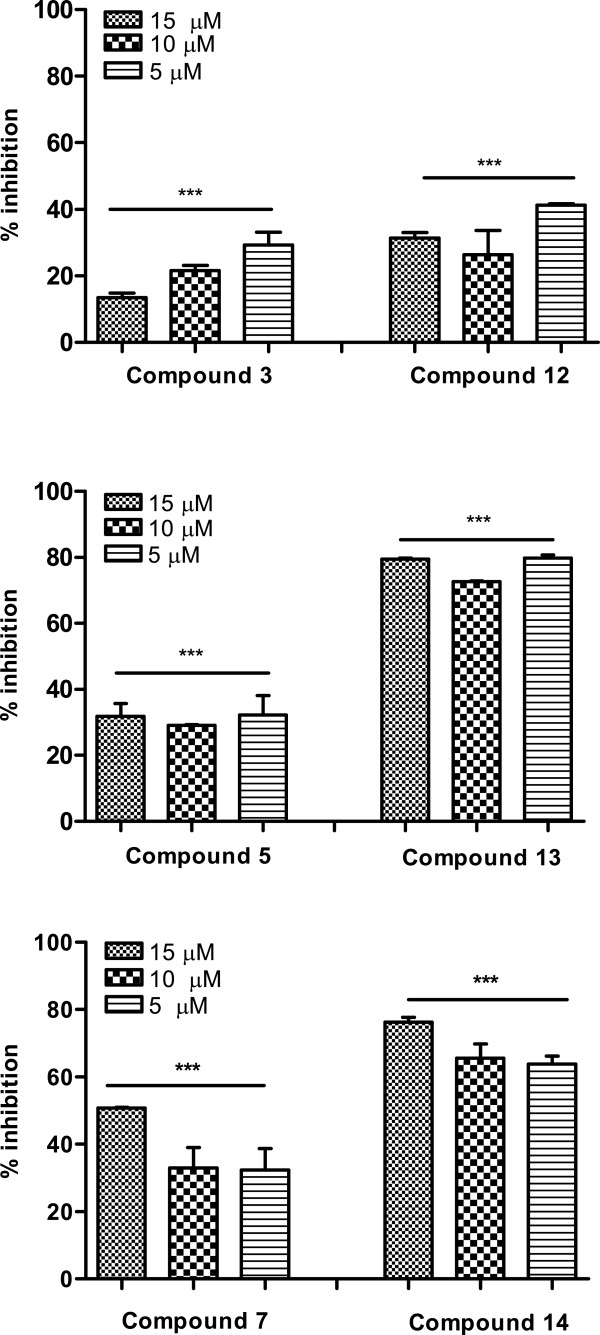
**Effect of alkyne intermediate structures (3, 5 and 7) and aminoquinoline/steroid conjugates (12, 13 and 14) on intracellular amastigotes. **Peritoneal macrophages previously infected with *L. major *promastigotes in the stationary growth phase were exposed to the compounds for 72 h. Results from two assays in duplicate are shown as percentage of growth inhibition in relation to untreated control. All results were significant (*** *p* < 0.0001).

Although the promastigotes of the *Leishmania* genus are used for screening of compounds, this assay must be considered as preliminary because: this stage of parasite is significantly more susceptible to drug-induced effects than amastigote, the amastigote are responsible for all clinical manifestations in humans and the intracellular amastigote model has been cited as the golden standard for *in vitro Leishmania* drug discovery research [33,34]. *L. major*-macrophage treated with the aminoquinoline/steroid compounds (**12**–**14**) showed a significant inhibitory effect against the intracellular amastigotes, as evidenced in Figure 4. Addition of a steroid group to aminoquinoline molecules again enhanced the biological activity of the compounds. Results showed that the compounds **13** and **14** showed the best antiproliferative effects on intracellular amastigotes, inhibiting between 64 and 80% of the parasite burden. These assays were performed in concentrations above those toxic for murine macrophages.

Antileishmanial and anti-MTB results confirm the importance of steroid groups such cholic acid acting as carriers. The cholic acid-derived carriers can possibly increase the solubility in physiological conditions and it could lead to increased cell permeability due to the amphiphilic character of the molecule and could function as an ionophore [22]. Further *in vivo* mouse model studies could better elucidate the role of bile acid derivatives as carriers.

## Conclusions

Regioselective synthesis of the novel aminoquinoline/steroid conjugates was achieved in very high yield. Addition of a steroid group to aminoquinoline molecules enhanced the anti-MTB activity, having lower MICs than some drugs commonly used to treat TB. For antileishmanial assay, the aminoquinoline/steroid conjugates demonstrated a significant activity against promastigote and amastigote forms of *L. major*.

## Abbreviations

BHI: Brain heart infusion; FBS: Fetal bovine serum; IC_50_: Concentrations inhibiting parasite growth by 50%; MIC: Minimum inhibitory concentration; MTB: Mycobacterium tuberculosis; TB: Tuberculosis.

## Competing interests

The authors declare that they have no competing interests.
